# Paracrine effects of TLR4-polarised mesenchymal stromal cells are mediated by extracellular vesicles

**DOI:** 10.1186/s12967-016-0794-z

**Published:** 2016-02-02

**Authors:** Marie-Theres Zeuner, Ketan Patel, Bernd Denecke, Bernd Giebel, Darius Widera

**Affiliations:** Stem Cell Biology and Regenerative Medicine Group, Reading School of Pharmacy, University of Reading, Whiteknights Campus, PO Box 226, Reading, RG6 6AP UK; School of Biological Sciences, University of Reading, Whiteknights Campus, Reading, UK; Freiburg Institute for Advanced Studies (FRIAS), University of Freiburg, Albertstr. 19, 79104 Freiburg, Germany; Interdisciplinary Center for Clinical Research Aachen (IZKF Aachen), RWTH Aachen University, Aachen, Germany; Institute for Transfusion Medicine, University Hospital Essen, University Duisburg-Essen, Essen, Germany

**Keywords:** TLR4, LPS, Exosomes, Microvesicles, Extracellular vesicles, MSCs, Preconditioning

## Abstract

Mesenchymal stromal cells (MSCs) are adult stem cells able to give rise to bone, cartilage and fat cells. In addition, they possess immunomodulatory and immunosuppressive properties that are mainly mediated through secretion of extracellular vesicles (EVs). In a previous issue of Journal of Translational Medicine, Ti and colleagues demonstrated that preconditioning of MSCs with bacterial lipopolysaccharides results in secretion of EVs that can polarise macrophages towards anti-inflammatory M2 phenotype. Moreover, the authors suggest that EVs of ​lipopolysaccharide (LPS)-treated MSCs are superior to EVs of untreated MSCs concerning their ability to support wound healing. Our commentary critically discusses parallel efforts of other laboratories to generate conditioned media from stem cells for therapeutic applications, and highlights impact and significance of the study of Ti et al. Finally, we summarise its limitations and spotlight areas that need to be addressed to better define the underlying molecular mechanisms.

Multipotent MSCs can be easily raised from a variety of adult human tissues and organs, including bone marrow and adipose tissue [1], and possess immunomodulatory capabilities affecting the majority of immune cells [2]. As of Nov 2015, more than 540 clinical trials utilising MSCs have been registered in the database clinicaltrials.org [3].

It turns out that the therapeutic benefit of MSC-administration revealed in different proof of concept and clinical studies (for review see [4]) is frequently connected to paracrine/endocrine effects rather than to effects driven by the engraftment of MSCs into affected tissues and differentiation towards lost cell types [5–9].

Related to their proposed paracrine mode of action, several pre-clinical reports and a clinical treatment attempt of a Graft-versus-host disease (GvHD) patient provided evidence that MSCs exert their therapeutic functions via extracellular vesicles (EVs), such as exosomes and microvesicles [10–14]. Indeed, direct comparisons in mouse models of acute kidney failure and ischemic stroke revealed that MSCs and MSC-EVs exert comparable therapeutic effects [11, 12].

By definition, exosomes are small EVs (~70–150 nm) of endosomal origin, while microvesicles (100–1000 nm) bud from the plasma membrane [15, 16]. With the current technologies, nano-sized microvesicles and exosomes can hardly be discriminated at the experimental level. Consequently, the International Society of Extracellular Vesicles (ISEV) recommended to use, at least at the experimental level, the term EV rather than exosomes and microvesicles [17]. EVs are found in all body fluids and are secreted by almost all cell types, under physiological and pathological situations. Notably both, the content (a specific combination of different proteins, lipids, microRNAs as well as a small portion of mRNAs) and immunomodulatory features of EVs are cell type and context-dependent [15, 16, 18–20].

Toll-like receptors (TLRs) are transmembrane receptors which play pivotal roles in the innate immune system by recognizing a wide spectrum of pathogen-associated molecules or pathogen-associated molecular patterns (PAMPs) as well as endogenous danger-/damage-associated molecular patterns (DAMPs) [21]. The toll-like receptor 4 (TLR4) recognises bacterial lipopolysaccharides (LPS) that depending on their chemotype can induce pro-inflammatory or immunosuppressive effects [22]. Importantly, also DAMPs are able to act as both, pro-inflammatory and immunosuppressive stimuli (reviewed in [23]). The binding of a ligand to TLR4 results in the activation of both, MyD88-dependent and independent signalling pathways. The MyD88-dependent signalling culminates in nuclear translocation of the pro-inflammatory transcription factor NF-κB dimers (p50 and p65) and increased expression and secretion of pro-inflammatory cytokines (e.g., TNF-α), whereas an active MyD88-independent pathway results in activation of IRF3 and synthesis of the anti-viral and anti-inflammatory interferon-β [24].

MSCs express functional TLR4 [25, 26] but the consequences of its activation on proliferation, differentiation and migration seem to be largely dependent on the chemotype, purity and concentration of the ligand in addition to the duration of the exposure (reviewed in [27]). Besides such direct effects, MSCs can be efficiently polarised into a pro-inflammatory and anti-inflammatory phenotype by ligands of TLRs leading to respective changes in their immunomodulatory properties [28]. It has been recently reported that preconditioning of adipose-derived MSCs with low concentration of LPS (0.5 ng/ml, unspecified chemotype) improves the regenerative effects of MSCs-secretome in experimental hepatectomy in mice [29].

Here, we comment on the original research article by Ti and colleagues published recently in Journal of Translational Medicine [30]. In their study, the authors preconditioned umbilical cord-derived human MSCs with 100 ng/mL LPS (chemotype and purity not specified) for 48 h followed by collection of the supernatant and isolation of EVs via ultracentrifugation. They provide evidence that EVs of preconditioned MSCs are able to induce polarisation of the THP-1 monocyte cell line towards the anti-inflammatory M2 phenotype demonstrated by significant reductions in the levels of pro-inflammatory cytokine mRNA (IL-6, IL-1 and TNF-α) and increased expression of anti-inflammatory transcripts IL-10 and TGF-β. Subsequently, Ti et al. were able to demonstrate that preconditioning of MSCs with LPS causes a change of EV miRNA cargo. They identified let-7b to be up-regulated most prominently among all of the 40 differentially expressed miRNAs. This is highly relevant since tumour suppressor miRNA let-7b is known to participate in regulation of cell proliferation and apoptosis and targets TLR4 [31, 32]. Similarly, in the study by Ti and colleagues, let-7b over-expression resulted in a significant decrease of TLR4 protein in THP-1 cells. Finally, in a rat model of diabetes, the application of EVs of preconditioned MSCs into cutaneous wound led to reduced infiltration of inflammatory cells and greatly improved the overall wound healing. Notably, EVs released by non-LPS treated MCS seem to transmit some beneficial effects in terms of tissue regeneration, albeit less pronounced than effects mediated by EVs released by LPS-treated cells. However, a major shortcoming of the study is the lack of quantitative data regarding the effects of EVs in the in vivo wound healing model hampering a direct comparison between the respective experimental groups. In general, diabetic wound healing models in rodents represent a potent tool in regenerative medicine and allow quantitative measurement of regeneration by assessing the epithelial gap and wound contraction [33]. Such quantitative parameters would greatly facilitate the evaluation of the effects of MSC-derived EVs on wound healing.

In summary, Ti and colleagues show that LPS modulates the secretome of umbilical MSCs. The authors demonstrate that EVs secreted by LPS-treated MSCs are anti-inflammatory and immunomodulatory. They further suggest that these effects are at least partly mediated by EV miRNA cargo. Finally, this study suggests that the EV fraction released from LPS-treated MSCs is enriched for the miRNA let-7b, which is known to target the TLR4/NF-κB signalling pathway.

Although this study has high translational potential, there are several open questions that need to be addressed in future. Firstly, the study does not include LPS-treated MSCs as a control group in the wound healing model. Ideally, a direct comparison of the effects of transplanted EVs and the parental cells should be performed.

Secondly, the fate of LPS-treated MSCs remains an unresolved question. Since MSCs themselves can be polarised into pro- and anti-inflammatory phenotypes [28, 34], it is of major interest whether the pro- or anti-inflammatory polarised MSCs secrete the anti-inflammatory EVs. In the present study, the stimulation of MSCs with LPS has been performed in serum-free medium. Notably, fetal calf serum (FCS) contains soluble CD14. As human MSCs lack the expression of CD14 [35, 36] that is believed to be necessary for induction of the MyD88 independent (anti-inflammatory) signalling pathway [37, 38], it could be presumed that most of the cells in this study underwent pro-inflammatory polarisation.

To investigate the ability of MSC-EVs to polarise macrophages, human monocytic cell line THP-1, originally isolated from peripheral blood of a 1-year-old male patient suffering from acute monocytic leukemia, has been applied [39]. Although widely used to study monocyte/macrophage functions, THP-1-derived macrophages differ from primary macrophages in terms of proliferation rate, expression pattern and notably also in terms of their sensitivity to LPS [40]. Thus, primary macrophages (e.g., derived from PBMCs) would represent a more appropriate cell population to test the EV-mediated polarisation.

Another major question to address is the role that let-7b plays in the process of diabetic wound closure. It would be of interest to determine whether the introduction of let-7b alone to the wound bed would sufficient to promote tissue regeneration by modulating the polarisation of macrophages. This would be an attractive therapeutic avenue since miRNA can be delivered to the lesion site using relatively safe vectors including the AAV family.

Another unresolved issue is the impact of different TLR4 ligands on the EV polarisation. In this context, it is noteworthy that the balance between the NF-κB signalling and the IRF3 activity is largely ligand-dependent [41]. Unfortunately, the study by Ti et al. does not provide information on the chemotype and purity of the LPS used, which both play a pivotal role in the downstream signalling (reviewed in [27]). In particular, standard LPS preparations activate TLR2 in addition to TLR4. In contrast to TLR4, TLR2 exclusively signals through the MyD88-dependent pathway and lacks the ability to activate IRF3. Thus, different LPS ligands induce fundamentally different downstream signalling cascades and could trigger different autocrine effects in MSCs.

Finally, since EVs contain a context-dependent and cell type specific combination of different proteins and lipids in addition to miRNAs, a holistic comparison of the cargo between EVs of LPS-treated and control MSCs could lead to better understanding of their regenerative properties.

## Conclusions

Beyond its immediate relevance for the biology of MSCs, the study by Ti et al. establishes a perspective for improvement of cell-free therapy concepts in translational regenerative medicine. In future, EVs of in vitro “programmed” MSCs through inflammatory or damage signals could represent a promising and realistic alternative to stem cell transplantation. To exploit this potential, however, future research needs to unravel in detail their mode of action. Prior to therapeutic use it will be important to establish if pro- or anti-inflammatory polarised MSCs secrete the beneficial EVs. In addition, it will be interesting to treat MSCs with defined ultrapure LPS of different chemo- and serotypes prior to EV isolation. Such polarisation of MSCs via appropriate inflammatory signals could allow generating EVs with either pro- or anti-inflammatory properties. Notably, regeneration of various tissue including skeletal muscles require both, pro-inflammatory signals that drive proliferation and migration of progenitors as well as anti-inflammatory signals that regulate their differentiation and survival [42].

## Abbreviations

AAV: adeno-associated virus
CD14: cluster of differentiation 14
DAMPs: damage associated molecular patterns
EVs: extracellular vesicles
FCS: foetal calf serum
IL: interleukin
IRF3: interferon regulatory factor 3
LPS: lipopolysaccharides
MSCs: mesenchymal stromal/stem cells
MyD88: myeloid differentiation primary response gene 88
NF-κB: nuclear factor ‘kappa-light-chain-enhancer’ of activated B-cells
PAMPs: pathogen-associated molecular patterns
TGF- β: tumor growth factor beta
TLR2: toll-like receptor 2
TLR4: toll-like receptor 4
TNF: tumour necrosis factor
